# Supplementary figures and images for: Revolutionizing Breast Reconstruction: The Rise of Hybrid Techniques
Source: Medicina (Kaunas). 2025 Aug 9;61(8):1434. doi: 10.3390/medicina61081434 (PMC12388044; doi:10.3390/medicina61081434)

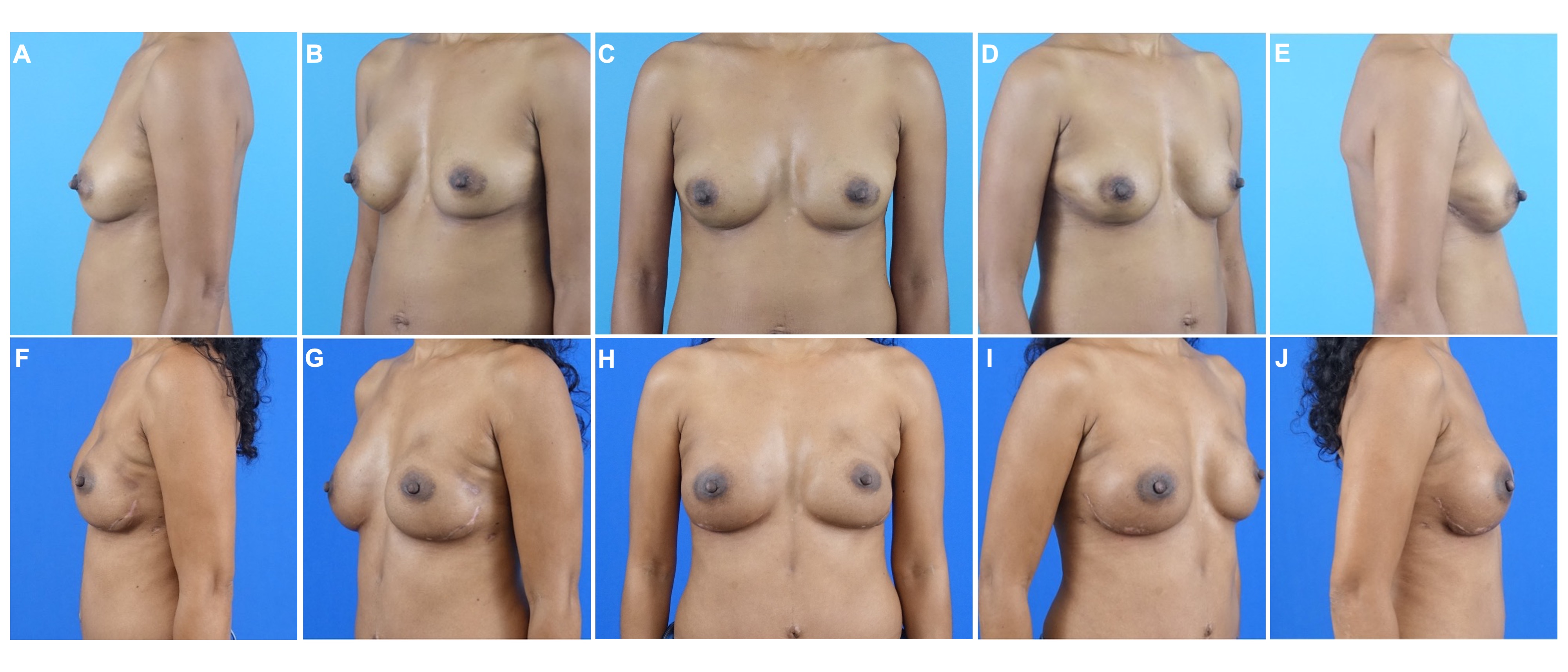

Supplement: Supplementary file 1 [file medicina-61-01434-s001.zip › Figure S1.jpeg]

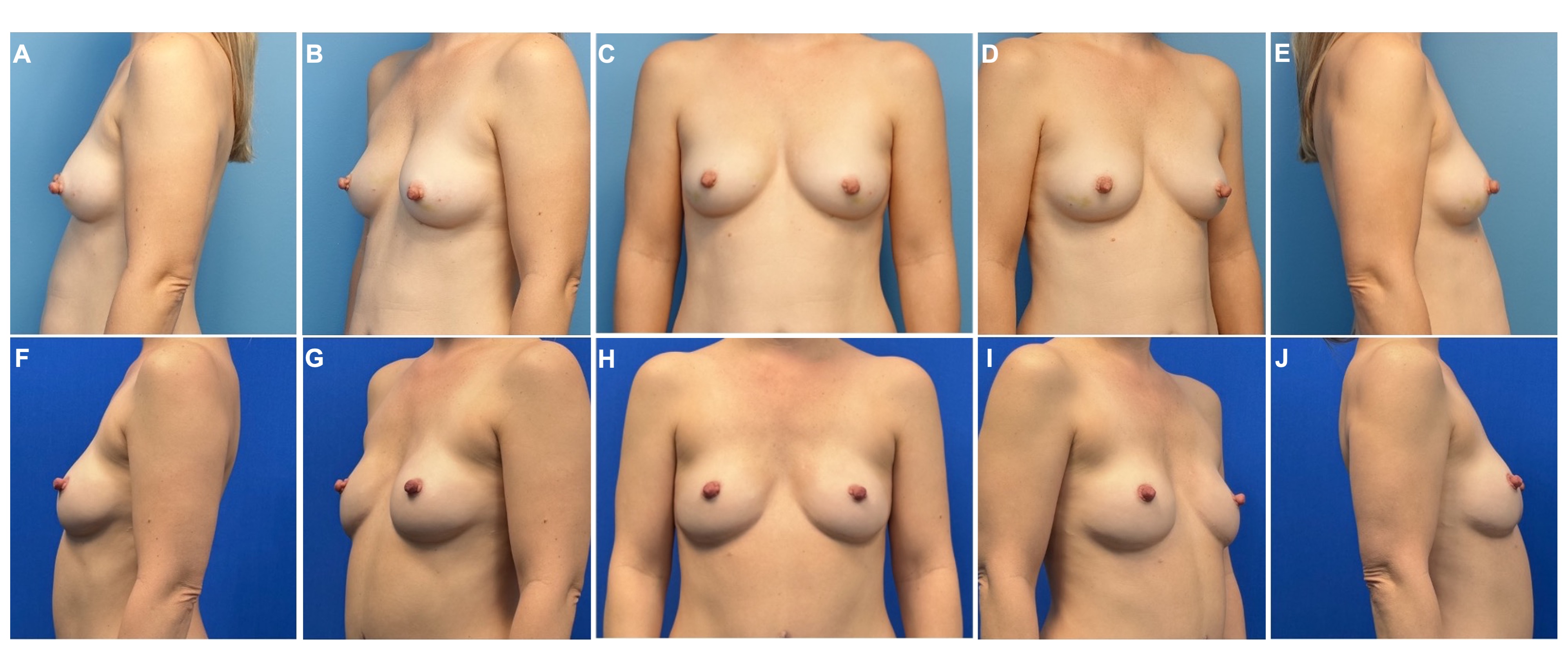

Supplement: Supplementary file 1 [file medicina-61-01434-s001.zip › Figure S2.jpeg]
